# Evaluating the efficacy of psychological therapies for generalised anxiety disorder in children and adolescents: A systematic review and narrative synthesis

**DOI:** 10.1002/jcv2.70056

**Published:** 2025-10-16

**Authors:** Lottie Shipp, Eleanor Leigh, Sakshi Rajesh, Polly Waite

**Affiliations:** ^1^ Department of Experimental Psychology University of Oxford Oxford UK; ^2^ Department of Psychiatry University of Oxford Warneford Hospital Oxford UK

**Keywords:** anxiety disorder, psychotherapy, randomised controlled trial, systematic review

## Abstract

**Background:**

Generalised Anxiety Disorder (GAD) is common in children and adolescents, and if not successfully treated, has negative consequences for their current and subsequent mental health. Whilst psychological therapies have previously been assessed in terms of their efficacy for a combination of anxiety disorders, no existing systematic reviews have evaluated GAD in isolation.

**Methods:**

To address this gap, a systematic review was undertaken in line with Preferred Reporting Items for Systematic Reviews and Meta‐Analyses guidance to explore the outcomes of psychological therapies for youth with a clinician‐assessed diagnosis of primary GAD. Searches of three databases (PsycINFO, Medline, and Embase) and two registers (Cochrane Central Register of Controlled Trials and ClinicalTrials) were conducted on 25th June 2024, and updated on 10th June 2025. Randomised controlled trials were eligible if they were published in a peer‐reviewed journal after 1994, and if data were available for children and adolescents with primary GAD only. We applied few exclusion criteria, with no limitations regarding treatment format, duration, or degree of therapist involvement.

**Results:**

Initial searches identified 7705 articles, but sufficient data were available for only 6 studies (*n* = 217 participants). Due to high heterogeneity, results were synthesised narratively rather than statistically. Evidence was limited in its quality and quantity, with few trials reporting outcomes or able to provide data at the level of individual anxiety disorders. Five studies reported higher rates of recovery in treatment groups relative to waitlist, and one reported greater remission for the control psychotherapy relative to a novel intervention. Given the lack of evidence, we were unable to assess cost‐effectiveness or the effects of therapeutic approach, age, depressive symptoms, or treatment adaptations for neurodevelopmental conditions.

**Conclusions:**

Psychological interventions may increase chances of remission for youth with GAD relative to no treatment, but the magnitude of the improvement is unknown. Further research and better data sharing practices are needed to determine which interventions are associated with the best treatment outcomes, and to identify for whom and under which circumstances they are most effective.

**Trial Registration:**

This review was pre‐registered on PROSPERO (CRD42024557665; https://www.crd.york.ac.uk/prospero/display_record.php?RecordID=557665).

## Key points

**What is known?**

- Generalised Anxiety Disorder (GAD) is a common anxiety disorder in youth, and is largely treated with psychological therapies.
- It is the exception rather than the norm for child and adolescent anxiety treatment trials to report results at the level of each individual diagnosis.

**What is new?**

- This systematic review is the first to assess diagnosis‐specific outcomes for children and adolescents with primary GAD.
- Whilst psychological therapies may increase chances of remission from primary GAD, we were unable to quantify the magnitude of their efficacy given the limited evidence base.

**What is significant for clinical practice?**

- Psychological therapies (in particular, those based on cognitive‐behavioural principles), are promising treatments for GAD in children and adolescents.
- Researchers should strive to analyse outcomes for the anxiety disorders separately rather than assuming that all diagnoses respond similarly to broad‐stroke transdiagnostic interventions.

## INTRODUCTION

Characterised by worry which is trans‐situational and excessive in nature (American Psychiatric Association, 2013), Generalised Anxiety Disorder (GAD) occurs in 0.7%–3.2% of 5–19 year‐olds (NHS Digital, 2018). It is one of the most common anxiety diagnoses amongst clinical child and adolescent populations (Waite & Creswell, 2014) and if left untreated, can trigger a cascade of detrimental long‐term effects (Hoffman et al., 2008; Pollard et al., 2023; Wittchen, 2002; Zugman et al., 2024). At a societal level, GAD is associated with a two‐fold increase in use of primary healthcare services (Wittchen, 2002), the highest per‐patient cost of any anxiety disorder (Hoffman et al., 2018), and the greatest use of emergency departments (Revicki et al., 2012). At an individual level, GAD is related to poorer quality of life and impairment that is comparable in magnitude to that associated with major depressive disorder (Hoffman et al., 2008)—yet it remains poorly recognised across the lifespan. This is particularly the case for youth (Dillon‐Naftolin, 2016; Keeton et al., 2009), with studies showing that teachers are less likely to identify anxiety problems in children with GAD compared to those with social anxiety disorder (Dadds et al., 1997). This may be because unlike the other anxiety diagnoses, GAD is not defined by overt behaviours (American Psychiatric Association, 2013), but is instead characterised by cognitive and somatic symptoms that may go unnoticed despite causing considerable distress. Furthermore, it is vital to study GAD in its own right given differences that set it apart from the other anxiety disorders—for instance, generalised rather than monothematic content (Cho et al., 2019), and its classification as a ‘distress disorder’ in the Hierarchical Taxonomy of Psychopathology framework (HiTOP; Kotov et al., 2017). These differences may have implications for treatments that target a range of anxiety diagnoses.

Most psychological interventions for youth anxiety have foundations in Cognitive‐Behavioural Therapy (CBT; Beck, 1976, 1985), and many are designed to target the common features of multiple anxiety disorders (e.g., ‘Coping Cat’; Kendall, 1994). Therapeutic components differ somewhat from treatment to treatment, but often include identification of anxiogenic cognitions and behaviours, relaxation training, cognitive restructuring, and progression through a graded exposure hierarchy (Kendall, 1994; Podell et al., 2010). These can be described as ‘transdiagnostic’ anxiety therapies, as their target processes span multiple disorders. In this review, the terms ‘transdiagnostic’ and ‘generic’ are used interchangeably. Other interventions tailor their therapeutic strategies to the profile of each individual anxiety diagnosis (i.e., ‘disorder‐specific’). In the context of GAD, these treatments aim to reverse the processes that maintain pathological worry—for example, dysfunctional metacognitions (Walczak et al., 2021), aversive attitudes and responses to uncertainty (Wahlund, Andersson, et al., 2020; Wahlund, Jolstedt, et al., 2020), cognitive avoidance, and negative problem orientation (Perrin et al., 2019). In general, recovery is achieved when worry is no longer used excessively to avoid or cope (Borkovec et al., 2007; Dugas et al., 1998; Gústavsson et al., 2021; Mennin et al., 2002; Roemer & Orsillo, 2002) and is reframed without positive connotations (Wells, 1995).

Currently, it is standard reporting practice to group the anxiety subtypes under the same taxonomic umbrella—that is, combining results into one statistic rather than evaluating outcomes on a diagnosis‐by‐diagnosis basis (Oldham‐Cooper & Loades, 2017; Reynolds et al., 2012). Systematic reviews taking this approach have shown that current psychological therapies lead to remission from the primary anxiety disorder for approximately half of children and adolescents (James et al., 2020; Lenz, 2015; Reynolds et al., 2012). Whilst informative, this monolithic treatment of the anxiety disorders limits our understanding of each in its own right. Preliminary evidence suggests that recovery rates differ between diagnoses (Evans et al., 2021; Hudson, Keers, et al., 2015, Hudson, Rapee et al., 2015), with some intervention studies reporting that primary GAD is associated with superior post‐treatment outcomes relative to other anxiety disorders in youth (Hudson, Keers, et al., 2015, Hudson, Rapee et al., 2015) but others finding lower recovery rates at long‐term follow‐up (Thirlwall et al., 2017). However, no existing reviews of treatment outcomes have focussed only on children and adolescents with GAD. Consequently, we do not yet know *which* psychological interventions are successful, or for *whom* they are effective within this diagnostic group. It is likely that the lack of clarity within research has contributed to the absence of clear clinical standards—for example, in the UK, guidelines for the management of GAD do not include recommendations for treating children and adolescents (National Institute for Health and Care Excellence, [NICE], 2020).

Furthermore, whether disorder‐specific treatments are more effective than transdiagnostic anxiety interventions for children and adolescents with GAD is currently unknown. An existing meta‐analysis for all anxiety disorders in youth (in addition to PTSD and OCD) reported larger effect sizes for disorder‐specific over generic psychotherapies—however, no interventions targeted only to GAD were identified (Reynolds et al., 2012). A more recent review by James et al. (2020) identified only two GAD‐specific studies (a mere 2.3% of the total number of included trials) and was unable to make conclusions regarding particular treatment approaches for individual disorders.

Participant‐level characteristics, such as age and depressive symptoms, may also influence treatment outcomes—for example, some studies find poorer remission from mixed anxiety disorders for adolescents compared to younger children (Ginsburg et al., 2011). However, others report no effect of participant age (Bennett et al., 2013), or the reverse pattern with greater remission in adolescents (Reynolds et al., 2012); yet notably, no studies have assessed GAD only. Furthermore, co‐occurring depressive symptoms have been linked with poorer treatment outcomes for childhood anxiety disorders in general (Hudson et al., 2013, Hudson, Keers, et al., 2015, Hudson, Rapee et al., 2015; Walczak et al., 2018). This relationship could become stronger when the focus is narrowed to GAD, given its closer relationship with depression than the other anxiety disorders (American Psychiatric Association [APA], 2013). However, this has not yet been formally reviewed.

Finally, the effects of neurodevelopmental conditions (e.g., autism spectrum conditions and ADHD) on treatment outcomes for youth GAD are currently unknown. Such conditions are associated with a greater risk of high anxiety symptoms (Brereton et al., 2006) and anxiety disorders (Kerns et al., 2021). Where cognitive‐behavioural treatments have been adapted to better suit the needs of neurodiverse youth, they appear to outperform standard control group (CBT) therapies in the context of mixed anxiety disorders (Kester & Lucyshyn, 2018; Wood et al., 2020). Yet once again, we are unaware of any research that has assess GAD in isolation.

### Research questions

1. How effective are psychological therapies for children and adolescents with GAD?
2. Do participant characteristics (age and baseline depression symptoms) relate to treatment outcomes?
3. Do treatment outcomes differ depending on whether the intervention is GAD‐specific or transdiagnostic, and whether it has been developed or adapted for children and adolescents with neurodevelopmental conditions?
4. How cost‐effective are current psychological therapies for GAD?

## METHODS

### Search strategy and selection criteria

This review followed the Preferred Reporting Items for Systematic Reviews and Meta‐Analyses (PRISMA) guidelines (Page et al., 2021; Rethlefsen et al., 2021) and was prospectively registered on PROSPERO (CRD42024557665). See Table 1 for the full inclusion criteria, and Supporting Information S1 for the PRISMA checklist.

**TABLE 1:** Inclusion criteria.

Criteria	Specification
Population	Children and adolescents (mean age of study population ≤ 18 years) with a clinician‐assessed primary diagnosis of GAD according to DSM‐IV or DSM‐5 criteria (American Psychiatric Association, 1994, 2013).
Intervention	Any targeted psychological therapy, defined as an intervention that aimed to reduce emotional distress by changing thoughts, feelings, and behaviours (or one's relationship to these) delivered directly to the child or adolescent.
Comparator	Any passive or active control condition. Active conditions were defined as treatments which, although plausible, did not meet full standard for psychological therapy (e.g., supportive listening), or those that were explicitly or implicitly identified as the control condition (e.g., by way of a directional hypothesis; Reynolds et al., 2012).
Outcome	Primary: Absence of a diagnosis of primary GAD at post‐treatment. Secondary: Severity of worry and/or anxiety symptoms at post‐treatment.
Specifications	Article published in English in a peer‐reviewed journal from 1994 onwards.
Setting	Any setting.
Study design	Randomised controlled trials.

Abbreviations: DSM, Diagnostic and Statistical Manual of Mental Disorders; GAD, Generalised Anxiety Disorder.

We restricted our synthesis to studies that used DSM‐IV or DSM‐5 criteria of GAD. This limited our search to articles published after 1994, when the uncontrollability of worry was established as the hallmark characteristic of GAD which was applied to children who would previously have been diagnosed with overanxious disorder.

Treatments delivered directly to the young person via any delivery format, of any duration, and with any degree of therapist involvement were included (i.e., from unguided self‐help to therapist‐led). However, studies assessing Attention Bias Modification were excluded, as this approach uses therapeutic techniques that were deemed to be incomparable with other eligible therapies. Interventions that selected participants on the basis of anxiety co‐occurring with a physical health condition or other psychological disorder were also excluded. Whilst interventions that had been adapted for children and adolescents with neurodevelopmental conditions were eligible, these were grouped separately.

A systematic search was conducted by LS on 25th June 2024 via three electronic databases (PsycINFO, Medline, and Embase) and two registers (Cochrane Central Register of Controlled Trials and ClinicalTrials). Additionally, forwards and backwards citation searching of the reference lists of eligible studies and key reviews was conducted (Baker et al., 2021; Cardy et al., 2020; Davis et al., 2011; Dülsen & Baumeister, 2024; Ewing et al., 2015; James et al., 2020; Oldham‐Cooper & Loades, 2017; Zhou et al., 2018). Grey literature was not included given concerns regarding quality, and to ensure the review reflected the most up‐to‐date evidence base, all searches were repeated on 10th June 2025 by LS. The same strategy as the original search was used, with the only difference being the date filter which was set such that only publications from 2024 to 2025 were retrieved.

The search strategy captured the following concepts linked with the Boolean operator ‘AND’: (1) GAD (‘*anxiety*’ OR ‘*anxious*’ OR ‘*GAD*’ OR ‘*worry*’), (2) children and adolescents (‘adolescen*’ OR ‘*youth*’ OR ‘*child*’ OR ‘teenage*’ OR ‘p?edia*’), (3) psychological therapy (‘therap*’ OR ‘treatment*’ OR ‘psychotherapy*’ OR ‘intervention*’ OR ‘*mindfulness*’ OR ‘*counselling*’ OR ‘*CBT*’ OR ‘*IPT*’ OR ‘*ACT*’ OR ‘*EFT*’), and (4) RCTs (‘*randomi#ed*’). The search strategy comprised only the terms that determined a study's eligibility for inclusion in the review, rather than addressing the additional concepts covered by our latter three research questions (neurodevelopmental conditions, depressive symptoms, cost‐effectiveness, etc.). This kept the search as broad as possible, minimising the risk of missing any studies relevant to the first and main research question. See Supporting Information S1: Appendix S1 for the full search strategy.

### Data selection, extraction, and quality rating

All studies were initially screened based on their titles and abstracts by LS using Rayyan software (Ouzzani et al., 2016), and 20% were independently double‐screened by SR. This process was conducted conservatively, such that any potentially eligible studies were escalated to the next stage. Cohen's kappa indicated good inter‐rater reliability (Cohen's kappa = 0.70, 99% agreement), with only 17 conflicts which were all resolved through discussion. LS screened all full texts and a random sample of 20% was independently double screened by SR. Four conflicts were discussed and resolved with the wider supervisory team.

Data regarding the context of the study, participant demographics, nature of the intervention, and results (including any adverse events) were extracted by LS using a standardised form. A random sample of 20% was independently extracted by SR, with cross‐checking revealing 100% agreement. For the secondary outcomes, questionnaire hierarchies were developed a‐priori. Consequently, if studies adopted multiple measures for the same construct, only one scale was selected according to the pre‐specified order of priority (see Supporting Information S1: Appendix S2). Intention‐To‐Treat (ITT) outcome data was extracted following an ‘as‐randomised’ approach (Chaplin & Dwan, 2024). Aligning with Baker et al. (2021), where only completer results were available, missing data were imputed using conservative ‘worst‐case’ criteria (i.e., assuming remission for drop‐outs from the control condition but not the intervention condition).

The Cochrane Collaboration Risk of Bias 2 tool (RoB2; Sterne et al., 2019) was chosen for its well‐validated framework and specificity to randomised trials (Kolaski et al., 2024). LS conducted all RoB2 assessments, with a random sample of 20% independently completed by SR. Agreement between raters was high (83%), with only two discrepancies which were both resolved via discussion. LS also applied the Grading of Recommendations, Assessment, Development and Evaluations tool (GRADE; Schünemann et al., 2019). No funnel plot was constructed to assess publication bias as these may be misleading when a search returns fewer than 10 studies (Dalton et al., 2016; Terrin et al., 2005).

### Data synthesis and analysis

Cochrane Collaboration guidance (Higgins et al., 2019) informed our data synthesis. Meta‐analysis was deemed unsuitable given the scarcity of eligible studies and high heterogeneity (McKenzie & Brennan, 2019), so a narrative synthesis was conducted following Popay et al.’s (2006) four‐element framework and the synthesis without meta‐analysis (SWiM) guidelines (Campbell et al., 2020; see S6 for completed SWiM checklist). Log odds ratios were calculated for the primary outcome, such that positive values indicated greater chance of remission from primary GAD at post‐treatment in intervention over control conditions. For secondary outcomes (severity of anxiety/worry symptoms at post‐treatment), we calculated effect sizes (Cohen's *d*). These were interpreted in line with Cohen's (1988) guidelines (small = 0.2, medium = 0.5, large = 0.8).

## RESULTS

**FIGURE 1:**
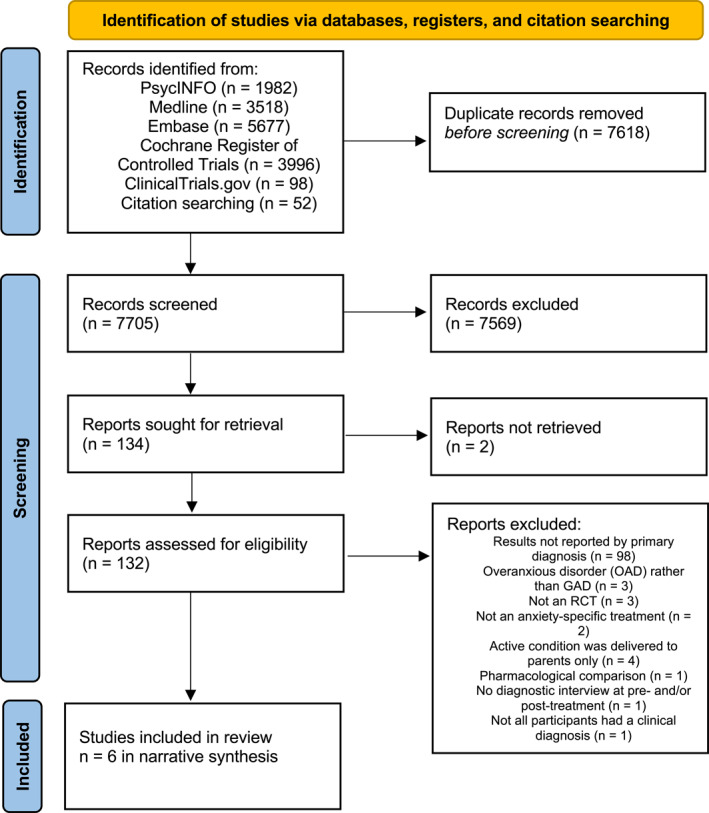
PRISMA flow/study selection. PRISMA, Preferred Reporting Items for Systematic Reviews and Meta‐Analyses.

Original searches were conducted by LS on 25th June 2024, and identified 15,323 records. Once duplicates were removed and initial screening of 7705 titles and abstracts was completed, 134 full texts were sought. Two were unavailable and authors did not reply to email requests; consequently, 132 texts (relating to 24 separate studies) were examined in full. See Figure 1 for the PRISMA flow diagram of the initial search.

Further information was required for 23 of the 24 studies, so corresponding authors were contacted via email. Ten (43%) did not reply or emails were undelivered, and 11 (49%) replied to our requests but were unable to share data (e.g., due to restrictions imposed by institutional or ethical policies, files no longer being in existence, or lack of time to source information). Two authors (9% of those contacted) provided necessary data. Subsequently, six studies were included in the narrative synthesis. An additional five studies provided relevant data for their whole sample but lacked information at the level of individual diagnoses and were therefore summarised in a table (see Supporting Information S1: Table S1) rather than forming part of the main synthesis.

A pilot trial by Ginsburg and Drake (2002) was identified as potentially eligible; however, diagnostic information was missing for those who completed less than two treatment sessions. As it was unknown whether these participants met criteria for primary GAD, we were unable to include the study in our synthesis.

Updated searches were conducted by LS on 10th June 2025 to identify any newly published studies, and ensure the review's relevance and accuracy. After removing duplicates, LS screened 1285 titles and abstracts, and identified 15 potentially eligible articles. The full texts of these articles were subsequently screened; however, all but one were deemed ineligible (*n* = 2 did not allocate specific anxiety diagnoses, *n* = 3 were not RCTs, *n* = 6 did not report disorder‐specific outcomes, *n* = 2 did not conduct post‐treatment diagnostic assessments, and *n* = 1 included youth with either anxiety or depressive disorders). The single remaining article, published by Skumsnes et al. (2024), provided additional data on a trial that was included in the review from the first search (Wergeland et al., 2014). The author's findings were integrated alongside the original results provided by Wergeland et al. (2014).

### Study characteristics

The narrative synthesis comprised data from a total of 217 participants (*n* = 128 in the treatment conditions and *n* = 89 in the control conditions). Trials were conducted in the UK (Perrin et al., 2019), US (Clementi & Alfano, 2020), Australia (Holmes et al., 2014), Norway (Villabø et al., 2018; Wergeland et al., 2014), and Germany (Goldbeck & Ellerkamp, 2012). Three took place in university clinics (Clementi & Alfano, 2020; Goldbeck & Ellerkamp, 2012; Holmes et al., 2014), and the remainder occurred in routine treatment settings (Perrin et al., 2019; Villabø et al., 2018; Wergeland et al., 2014). Two studies (33%) reported no adverse events related to trial activities, whereas the remaining four (67%) did not report on adverse events. Participants' average age ranged from 9.0 to 13.4 years (range 6–18 years).

In line with our research questions, studies were grouped according to the nature of the intervention (i.e., GAD‐specific or transdiagnostic) and presented in tables in order of risk of bias. Included transdiagnostic treatment trials published results separately for each primary diagnosis (Goldbeck & Ellerkamp, 2012), or authors responded to requests for GAD‐specific data (Villabø et al., 2018; Wergeland et al., 2014).

**FIGURE 2:**
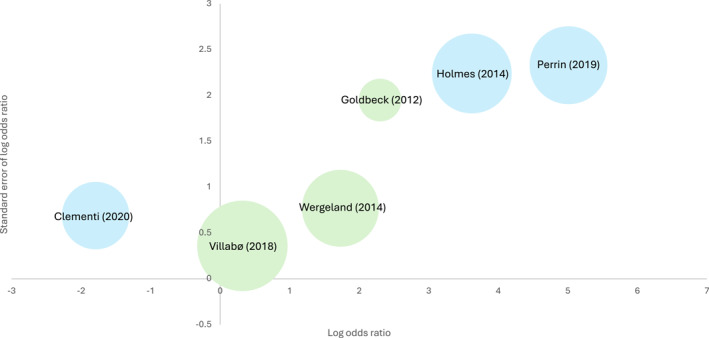
Log odds ratios of remission from primary GAD at post‐treatment. This graph displays the log odds ratio of remission for each study included in the narrative synthesis, plotted against standard error of the log odds ratio. A positive log odds ratio indicates results in favour of the intervention over the control condition. The size of each bubble represents the study sample size (i.e., number of participants who met diagnostic criteria for primary GAD at baseline). Blue = GAD‐specific psychological therapies; green = transdiagnostic psychological therapies. GAD, Generalised Anxiety Disorder.

Across all six studies, the number needed to treat was 3.19; put simply, approximately three children and adolescents needed to receive treatment to prevent one additional negative outcome. See Table 2 for a summary of main findings, and Figure 2 for a graphical display of primary outcomes. It is noted that the latter is presented simply to show the heterogeneous spread of results, and should not be interpreted as a statistical synthesis.

**TABLE 2:** Summary of study characteristics.

Study	Psychological therapy	Control condition	Primary outcome: Log odds ratio of remission (SE)	Secondary outcome: Anxiety symptom measure	Secondary outcome: Post‐treatment anxiety severity (Cohen's d (95% CI))	Secondary outcome: Worry symptoms at post‐treatment (PSWQ‐C)	Proportion drop‐out: Intervention/control	Cost‐effectiveness	Treatment acceptability	Neurodiversity
Perrin (2019)	CBT	WLC	5.01 (2.33)	CSR	2.11 (1.34 to 2.88)—ITT	2.05 (1.28–2.81)—PSWQ‐C, ITT	20%/0%	Not measured	Measured with an adapted version of the credibility and expectation questionnaire. Treatment was judged to be logical, likely to succeed and recommendable to a friend. Therapists were rated as warm and engaged.	One participant had ADHD and one had a motor disorder. Children and adolescents with autism were excluded at screening. The presence of moderate to severe learning difficulties was an exclusion criteria.
Holmes (2014)	Cognitive therapy	WLC	3.62 (2.24)	CSR	2.21 (1.44 to 2.98)—Completer only	0.71 (0.09–1.34)—PSWQ‐C, completer only	15%/14%	Not measured	Treatment was rated as moderately satisfactory by children and parents (using an 8‐item author‐developed questionnaire).	Nine participants had ADHD. The presence of pervasive developmental disorders, intellectual handicap, or learning disability was an exclusion criteria.
Clementi (2020)	Targeted behavioural therapy	Coping Cat	−1.79 (0.69)	SCARED‐C	−0.63—ITT	Not measured	27%/34%	Not measured	Not measured	One participant had ADHD. The presence of pervasive developmental disorders was an exclusion criteria.
Wergeland (2014)	CBT	WLC	1.73 (0.77)	N/A	Data not available for primary GAD sample	Not measured	Unknown for primary GAD only	Not measured	Not measured	Not known for GAD sample. Exclusion criteria included ‘pervasive developmental disorder, psychotic disorder, and/or mental retardation’ (p.3).
Villabø (2018)	CBT	WLC	0.32 (0.36)	CSR	0.50 (−0.15 to 1.15)—ITT	Not measured	21%/25%	Not measured	Measured with the questionnaire for evaluation of treatment (child, parent, and therapist reports). For the mixed anxiety sample, overall treatment satisfaction was good.	Two participants had ADHD. The presence of pervasive developmental disorders was an exclusion criteria.
Goldbeck (2012)	Multimodal music therapy	TAU	2.30 (1.95)	N/A	Data not available for primary GAD sample	Not measured	Unknown for primary GAD only	Not measured	Not measured	Not known for GAD sample. Children with an IQ less than or equal to 80 were ineligible.

*Note*: Secondary effect size for Clementi (2020) was calculated from partial eta squared, and it was not possible to compute a confidence interval. Study name includes first author only.

Abbreviations: 95% CI, 95% confidence interval; CBT, Cognitive Behavioural Therapy; CSR, Clinician Severity Rating; GAD, Generalised Anxiety Disorder; ITT, Intention To Treat; PSWQ‐C, Penn State Worry Questionnaire for Children; SCARED‐C, Screen for Child Anxiety Related Disorders; SE, Standard Error; TAU, Treatment As Usual; WLC, Waitlist Control.

### GAD‐specific psychological therapies

The Anxiety Disorder Interview Schedule (ADIS‐C/P; Silverman & Albano, 1996) was used in all three GAD‐specific studies, and was conducted by independent, blind assessors. Clementi and Alfano (2020) reported demographic information only for treatment completers (*n* = 21, or 70% of those randomised), whereas data were available for the ITT sample for the other two studies. This resulted in demographic profiles for *n* = 103 of the total *n* = 112 participants randomised across all three studies. Of these 103, 49% had co‐occurring social anxiety disorder and 40% had separation anxiety disorder. Depression diagnoses were not reported by Clementi and Alfano (2020), and *n* = 8 participants in the other two studies met criteria (10% of *n* = 82). Eleven percent of participants had ADHD, 60% were female, and none identified as non‐binary. Studies excluded youth with autism (Perrin et al., 2019), moderate to severe learning difficulties (Holmes et al., 2014; Perrin et al., 2019), intellectual handicap (Holmes et al., 2014), and/or pervasive developmental disorders (Clementi & Alfano, 2020; Holmes et al., 2014).

All GAD‐specific psychological treatments met Chambless and Hollon's (1998) criteria for robust treatment implementation, were based on a CBT framework, and required 10–16 face‐to‐face sessions. Therapies were delivered in individual (Clementi & Alfano, 2020; Perrin et al., 2019) or group (Holmes et al., 2014) settings.

Two trials (Holmes et al., 2014; Perrin et al., 2019) assessed treatments that targeted the features of GAD outlined in Dugas et al.’s (1998) intolerance of uncertainty model and compared outcomes to a waitlist group. Holmes et al.’s (2014) intervention also targeted sleep difficulties and perfectionism, but in contrast to Perrin et al.’s (2019) treatment, did not include explicit in‐session exposure. Perrin et al. (2019) delivered a modified version of Dugas and Robichaud's (2007) adult treatment, whereas Holmes et al. (2014) assessed the youth‐specific No Worries! programme. Both interventions were designed to be appropriate for a younger population—for example, using a ‘Worry Beast’ metaphor to explain abstract concepts (Holmes et al., 2014).

Taking a different approach, Clementi and Alfano (2020) used an active control condition (Coping Cat) which was compared to individual ‘targeted behavioural therapy’ (TBT). This novel treatment incorporated elements of CBT, but given the link between youth GAD and sleep difficulties, dedicated four sessions to a sleep intervention. It should be noted, however, that their participants were not recruited on the basis of sleep problems. Instead (comparable to the other two studies), they were eligible solely due to their primary GAD diagnosis. See Table 3 for a summary of intervention and participant characteristics.

**TABLE 3:** Summary of intervention and participant characteristics (GAD‐specific therapies).

Study	Age (mean, range)	Total *n*	Severity of GAD/anxiety symptoms at baseline	Co‐occurring disorders	Level of depressive symptoms at baseline	Treatment components (child sessions)	Dose and duration	Clinician background	Level of parental involvement
Perrin (2019)	13.4 years (10–18 years)	40	Mean CSR (across conditions) = 6.8–6.9	Eighty‐three percent of participants had at least one co‐occurring disorder. The most common was separation anxiety disorder (25%), followed by social phobia (23%). 3% of participants had ADHD and 3% had a motor disorder.	Six participants had a diagnosis of major depression. Mean depression score (MFQ‐C) for the total sample = 27.0.	Worry awareness training, exposure to uncertainty, modification of dysfunctional beliefs about worry, problem‐solving training, imaginal exposure, and relapse prevention.	10 sessions for children over 10 weeks (duration of each session not reported).	Doctoral‐level clinical psychologists	Parents were invited to join the first session and the last 5–10 min of each subsequent session.
Holmes (2014)	9.64 years (7–12 years)	42	Mean CSR (across conditions, completer sample only) = 6.0–6.3	Participants met criteria for an average of 3.69 disorders. 64% had co‐occurring separation anxiety disorder, 80% had social phobia, 21% had ADHD and 14% had ODD. There were 37 specific phobia diagnoses across the sample.	Five participants had co‐occurring dysthymia/MDD. Mean parent‐reported internalising score (CBCL‐Int, across conditions, completer sample only) = 68–72.	Psychoeducation, relaxation training, techniques to target the key aspects of the intolerance of uncertainty model (intolerance of uncertainty, negative beliefs about worry, cognitive avoidance, and negative problem. Orientation), addressing sleep issues related to worry, and reducing perfectionism.	10 weekly sessions and 2 booster sessions for children (90 min each). 7 weekly sessions and 2 booster sessions for parents (90 min each). Total of 31.5 h per child.	Trained postgraduate students	7 parent sessions and 2 booster sessions which ran alongside child sessions.
Clementi (2020)	9.04 years (6–12 years)	30	Mean SCARED‐C score (across conditions, completer sample only) = 31–39	Social phobia was the most common co‐occurring disorder (30%), followed by separation anxiety disorder (13%), specific phobia (10%), and 3% each for panic disorder, ODD, ADHD, and enuresis.	No depression or dysthymia diagnoses reported. No use of self‐ or parent‐reported measures of depressive symptoms.	Psychoeducation, sleep education and intervention, problem‐solving, relaxation training, exposure to uncertainty, relapse prevention.	16 weekly sessions (60 min each). Total of 16 h per child.	Advanced doctoral students and postgraduate fellows	First 5 sessions and the final session involved both parent and child. The remaining sessions included a brief check‐in with the parent.

*Note:* Study name includes first author only.

Abbreviations: CBCL‐Int, Child Behaviour Checklist, Internalising Subscale; CSR, Clinician Severity Rating; GAD, Generalised Anxiety Disorder; MFQ‐C, Mood and Feelings Questionnaire, Child Version; SCARED‐C, Screen for Child Anxiety Related Disorders, child report.

All three studies used questionnaires to collect self‐ and/or parent‐reported symptoms of GAD and/or anxiety. A single study included the Short Mood and Feelings Questionnaire (SMFQ; Angold et al., 1995) to measure depression symptoms (Perrin et al., 2019), one evaluated broader internalising problems with the Child Behaviour Checklist (CBCL; Achenbach, 1999; Holmes et al., 2014), and one did not report on depressive symptoms (Clementi & Alfano, 2014). Only one study provided raw data for the ITT sample for both primary and secondary outcomes (Perrin et al., 2019). Holmes et al. (2014) provided remission information for the ITT sample, but raw scores on secondary measures were available only for the completer sample. Clementi and Alfano (2020) reported completer data only for both primary and secondary outcomes, from which we calculated remission rates for the ITT sample according to our pre‐registered procedure.

Of the two studies with waitlist controls, remission from primary GAD for treatment groups was 80% (Perrin et al., 2019) and 45% (Holmes et al., 2014), with both trials reporting 0% remission for waitlisted participants. In contrast, Clementi and Alfano (2020) found higher remission in the control condition (‘Coping Cat’; 80%) relative to the treatment group (TBT; 40%). Consequently, log odds ratios for remission from primary GAD at post‐treatment in intervention relative to control conditions ranged from −1.79 (Clementi & Alfano, 2020) to 5.01 (Perrin et al., 2019). Clementi and Alfano (2020) were the only researchers to report results in favour of the control treatment, reflecting the use of a well‐validated CBT intervention as a control condition rather than waitlist.

With respect to secondary outcomes, Holmes et al. (2014) and Perrin et al. (2019) reported significant reductions in clinician‐rated GAD severity and self‐reported worry. Baseline GAD severity was similar across both studies, dropping below the clinical range for the intervention but not control groups at post‐treatment with large effect sizes (Cohen's *d* = 2.21 and 2.11, respectively). In contrast, Clementi and Alfano (2020) used a broad self‐report anxiety measure (Screen for Child Anxiety Related Disorders, child report [SCARED‐C]). Baseline anxiety was high (mean scores of 38.94 and 30.69 in the intervention and control groups, respectively) relative to other studies which have reported SCARED‐C total scores of approximately 23 (Behrens et al., 2019; Caporino et al., 2017). Children's self‐reported anxiety reduced in both the TBT and Coping Cat groups for treatment completers (change in pre‐ to post‐treatment effect sizes of *d* = 1.53 for TBT and *d* = 0.52 for Coping Cat), but the group × time interaction was not significant and ITT data were unavailable.

All studies conducted follow‐up assessments 3 (Holmes et al., 2014; Perrin et al., 2019) or 6 months (Clementi & Alfano, 2020) after treatment, with drop‐out rates from 5% to 27%. Of the studies with waitlist controls, 85% and 89% of those who received CBT were in remission from GAD at the 3‐month assessment (Perrin et al., 2019; Holmes et al., 2014, respectively). These rates are substantially higher than Clementi and Alfano's (2020) reports of 47% in both the intervention and control conditions at 6‐month follow‐up (for the ITT sample), and for Holmes et al. (2014), represents a considerable increase from their post‐treatment remission rate of 45%. The authors suggest that this enhanced effect at follow‐up may result from consolidation of therapeutic strategies in real‐life situations.

Of note, neither Perrin et al. (2019) nor Clementi and Alfano (2020) assessed the effects of baseline anxiety severity on treatment outcome. However, Holmes et al. (2014) reported the results of subsidiary analyses using completer data, which found no difference in baseline anxiety between children who met diagnostic criteria for GAD at post‐treatment compared to those who did not. Children who reached remission from all anxiety disorders following treatment had less severe GAD symptoms at baseline than those who retained at least one anxiety diagnosis, but these analyses were not reported for the ITT sample.

### Transdiagnostic anxiety psychological therapies

Three studies (see Table 4) assessed transdiagnostic anxiety therapies (Goldbeck & Ellerkamp, 2012; Villabø et al., 2018; Wergeland et al., 2014), with data extracted for the 105 participants with primary GAD. Two conducted ADIS‐C/P assessments (Villabø et al., 2018; Wergeland et al., 2014) and one used the German version of the KIDDIE‐SADS Present and Lifetime version (Goldbeck & Ellerkamp, 2012; Kaufman et al., 1996). Assessors were blind to treatment allocation in two trials (Goldbeck & Ellerkamp, 2012; Villabø et al., 2018) but not the third (Wergeland et al., 2014; although independent experts largely agreed with initial decisions, as demonstrated by kappa = 0.86 for the presence of GAD). Co‐morbidity data were available only for Villabø et al.’s (2018) trial. The most common co‐occurring diagnosis was social anxiety disorder (42%), followed by specific phobia (33%) and oppositional defiance disorder (11%).

**TABLE 4:** Summary of intervention and participant characteristics (transdiagnostic therapies).

Study	Age (mean, range)	Total *n* (*n* with primary GAD)	Severity of anxiety/GAD symptoms at baseline	Level of depressive symptoms at baseline	Co‐occurring disorders	Treatment components (child sessions)	Dose and duration	Clinician background	Level of parental involvement
Wergeland (2014)	11.0 years (8–15 years)	182 (39)	Mean CSR (across conditions, *n* = 37) = 6.8	Mean depression score (SMFQ‐C, *n* = 33) = 8.1	For *n* = 182, co‐occurring disorders included specific phobias (*n* = 18), other anxiety disorders (*n* = 9), depression (*n* = 21), ADHD (*n* = 10), ODD (*n* = 10), tic disorder (*n* = 12), and anorexia (*n* = 2).	Relaxation, identifying and challenging anxiogenic thoughts, problem‐solving and social support training, and exposure.	Children received 10 weekly sessions (60 min for ICBT and 90 min for GCBT) and 2 booster sessions. Parents received 2 separate sessions (group or individual depending on child's allocation), and attended 2 of their child's sessions.	Experienced clinical psychologists, clinical pedagogues, and clinical social workers.	Parents received two separate sessions, attended two of their child's sessions, and the last 15 min of every other session.
Villabø (2018)	10.5 years (7–13 years)	165 (54)	Mean CSR (across conditions) = 6.5	Two participants had diagnoses of dysthymia, and two had depression.	Co‐occurring anxiety disorders included separation anxiety disorder (*n* = 23), social or specific phobia (*n* = 18 each), ADHD (*n* = 11), OCD (*n* = 2), panic/agoraphobia (*n* = 1), PTSD (*n* = 1), ODD (*n* = 1), and enuresis (*n* = 1)	Psychoeducation about anxiety, exposure tasks, somatic management (including relaxation training), cognitive restructuring, and problem‐solving training.	Children received 12 sessions over 12 weeks (no duration given). Parents received 2 separate sessions. Those allocated to the group condition had 3 individual sessions prior to joining a group for the remainder of treatment.	Clinical psychologists, clinical social workers, psychiatry residents, and clinical pedagogues (69% had prior experience with CBT).	Parents received two separate sessions.
Goldbeck (2012)	9.94 years (8–12 years)	36 (12)	Of the total sample (*n* = 36), *STAIC‐T‐C* T‐score (across conditions) = 48–51	Of the total sample (*n* = 36), *n* = 2 had a diagnosis of depression. CDI T‐score (across conditions) = 48–54	11% of participants had a second anxiety disorder, and 3% had encopresis, ADHD or elective mutism.	Active and receptive music therapy combined with CBT techniques (psychoeducation, social skills training, exposure to anxiogenic stimuli, homework assignments, and relaxation training).	Children received 3 individual and 9 group sessions (60 and 100 min, respectively). Parents received 1 group and 2 individual sessions (100 and 50 min, respectively). Total of 22.83 therapist hours per child.	Qualified and trainee music therapists, and a clinical psychologist.	Parents had three separate sessions (1 group and 2 individual).

*Note*: Demographics presented for Goldbeck (2012) is for the whole study sample, whereas Villabø (2018) and Wergeland's (2014) demographic information is for only the subset of participants with primary GAD at baseline unless stated otherwise. Study name includes first author only.

Abbreviations: CBT, Cognitive‐Behavioural Therapy; CDI, Children's Depression Inventory; CSR, Clinician Severity Rating; GAD, Generalised Anxiety Disorder; SMFQ‐C, Short Mood and Feelings Questionnaire, Child Report; STAIC‐T‐C, State Trait Anxiety Inventory for Children‐Trait subscale, child report.

Two studies were conducted within community clinics, used a waitlist comparator, and delivered treatment in group and individual formats. For the purposes of this review, and in light of the lack of substantial differences in outcome between individual and group conditions, data for the two delivery formats were combined into a single ‘active’ group for both trials. The third study (Goldbeck & Ellerkamp, 2012) used Treatment As Usual (TAU) as the control condition (including a range of cognitive and behavioural interventions with an average of four sessions per participant).

The interventions delivered by Wergeland et al. (2014) and Villabø et al. (2018) met all criteria for robust treatment implementation (Villabø et al., 2018; Wergeland et al., 2014). However, the third study (Goldbeck & Ellerkamp, 2012) failed to conduct therapy integrity checks and therefore did not meet the last criterion. Well‐validated CBT treatments were assessed in two trials (FRIENDS and Coping Cat; Wergeland et al. (2014) and Villabø et al. (2018), respectively), whereas Goldbeck and Ellerkamp (2012) delivered a less established intervention that combined music therapy techniques with more traditional elements of CBT.

Wergeland et al. (2014) provided ITT remission rates from primary GAD at post‐treatment of 59% (*n* = 17) and 20% (*n* = 2) for the treatment and control conditions respectively, with a log odds ratio of 1.73. Additional data for this trial were provided in a follow‐up paper by Skumsnes et al. (2024), although their report combined data across the original intervention group and those who received treatment following the waitlist period (excluding *n* = 1 who dropped out whilst on the waitlist, and *n* = 1 who no longer met diagnostic criteria for GAD at the end of the waitlist period). Of the remaining 37 participants, 41% were recovered from all anxiety diagnoses at post‐treatment, and this increased to 62% at the 1‐year follow‐up assessment. Notably, rates were largely maintained at the 4‐year follow‐up assessment (59% in remission from all anxiety disorders); however, conclusions from this statistic are limited due by the lack of a waitlist comparison group at this time point. Furthermore, the mean CSR for this group dropped below the clinical range at post‐treatment (2.28, SD = 2.45) and remained within the non‐clinical range at both follow‐up assessments with means of 2.67 (SD = 2.13) and 1.15 (SD = 2.55) at 1‐ and 4‐year follow‐ups, respectively.

Villabø et al. (2018) provided raw data for the 44 treatment completers (81% of the original 54 with primary GAD who were randomised). From this, we calculated ITT data using the last outcome carried forward method, finding remission rates at post‐treatment of 58% and 50% following treatment or waitlist period respectively, and a log odds ratio of 0.32. Finally, Goldbeck and Ellerkamp (2012) reported higher remission rates for those who received multimodal music therapy compared to TAU (83% and 33% respectively, with a log odds ratio of 2.30). Secondary outcome data were only available for the primary GAD subgroups for Villabø et al. (2018), with Cohen's *d* = 0.50 for post‐treatment clinician‐reported anxiety symptoms.

### Studies reporting no moderating effect of initial primary diagnosis on outcomes

Additionally, we identified five RCTs of transdiagnostic treatments (Barrington et al., 2005; Cobham et al., 1998; Shortt et al., 2001; Silk et al., 2018; Suveg et al., 2018), in which results were not presented for each disorder individually, but statistical analyses identified no significant differences in treatment outcomes according to primary diagnosis. This was with the exception of Barrington et al. (2005), who reported that primary social phobia was associated with poorer treatment outcomes, but with no differences in outcomes between the other primary disorders. As such, we removed participants with primary social phobia from the overall results and present the outcomes only for the remaining primary diagnoses for this study.

Consequently, it may be assumed (albeit with less certainty) that remission rates for the mixed anxiety sample are representative of the results for the subset of participants with GAD. Whilst informative, this data is less precise and thus is discussed only briefly here and summarised in Supporting Information S1: Table S1.

These trials included 417 participants aged 6–14 years, of whom 238 (57%) had primary GAD at pre‐treatment. Only Shortt et al. (2001) employed a waitlist CBT; the others used an active CBT comparison (Cobham et al., 1998; Silk et al., 2018; Suveg et al., 2018) or TAU (Barrington et al., 2005). All treatments were grounded in CBT techniques, with four based on Coping Cat, Coping Koala, or the FRIENDS programs (Barrington et al., 2005; Cobham et al., 1998; Shortt et al., 2001; Silk et al., 2018) and one using emotion‐focused CBT (Suveg et al., 2018). Outcomes relevant to this review (i.e., remission data for which it was established that there was no moderating effect of initial primary diagnosis) were reported at post‐treatment (Cobham et al., 1998; Shortt et al., 2001; Silk et al., 2018; Suveg et al., 2018) and follow‐up (Barrington et al., 2005). One study reported a higher proportion of participants in remission from all anxiety disorders in the CBT relative to the treatment group (emotion‐focused CBT; Suveg et al., 2018); all other studies reported higher remission rates in intervention conditions. Recovery rates from all anxiety disorders varied from 35% to 84%, relative to 12%–75% in control groups. Log odds ratios ranged from −0.49 (Suveg et al., 2018) to 2.47 (Shortt et al., 2001).

### Risk of bias

**FIGURE 3:**
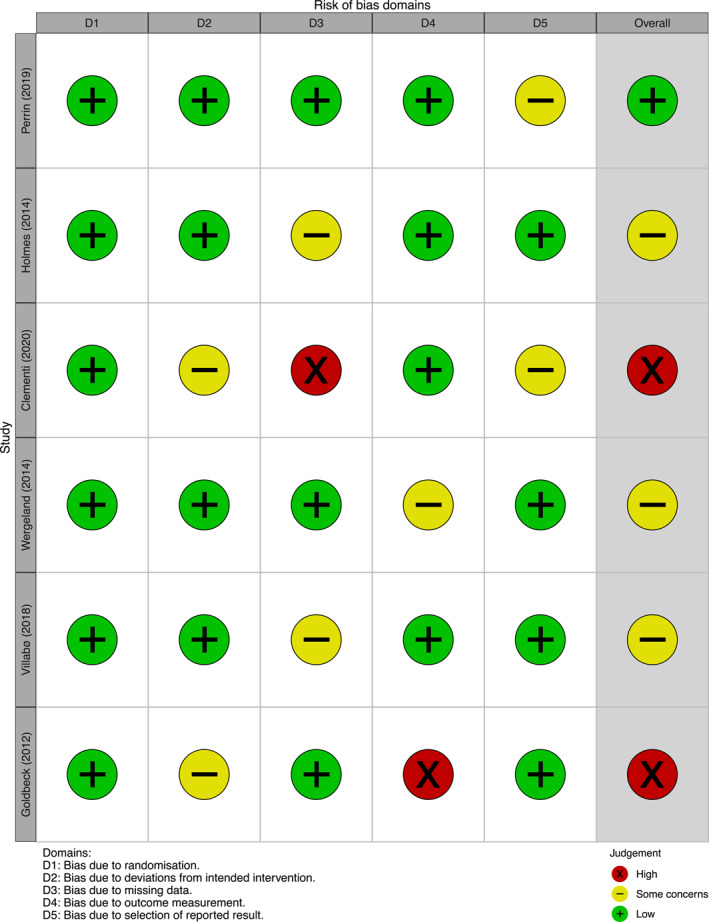
Risk of bias for the primary outcome.

Potential bias was detected across of four of the five domains (see Figure 3)—for instance, failure to report ITT results, awareness of assessors to participants' treatment allocation, and analysis plans that were either absent, registered prospectively, or lacking sufficient detail. For a detailed assessment of outcome reporting, please see Supporting Information S1: Appendix S3 for the outcome reporting bias in trials matrix (ORBIT; Kirkham et al., 2018).

The default overall judgement (i.e., equal to the highest risk of bias judgement in any one domain; Higgins et al., 2019; Sterne et al., 2019) was over‐ridden by the research team for one outcome. Some concerns were raised for Perrin et al. (2019) for the fifth domain, given its retrospective registration on ISRCTN. However, it was agreed that the overall risk of bias was low, as researchers followed standard practices in similar trials and thus results were unlikely to have been selectively reported.

### Certainty assessment

We followed guidance for the application of the GRADE approach to narratively synthesised data (Murad et al., 2017). There was considerable risk of bias, and the data were judged to be moderately indirect (e.g., study populations with a lack of neurodiversity), inconsistent, and imprecise. As such, whilst psychological therapy may result in a large increase in the likelihood of remission from primary GAD at post‐treatment, there remains a high level of uncertainty.

## DISCUSSION

This review summarises the current evidence for psychological therapies for children and adolescents with primary GAD. Our search identified 134 articles that reported on children and adolescents with GAD, but 98 of these did not include any diagnosis‐specific information, and over 90% of contacted authors were either unable to share data or were not reachable. Our narrative synthesis subsequently included only six trials, from which we conclude that psychological therapies may improve chances of remission from primary GAD at post‐treatment; however, the magnitude of this increase in unknown. Given insufficient data, we were unable to assess cost‐effectiveness or any of the participant or intervention characteristics of interest (transdiagnostic or GAD‐specific treatment approach, adaptation for neurodiversity, and participant age or depressive symptoms).

Extant GAD‐specific studies provide preliminary evidence for the efficacy of targeted interventions compared to no treatment. With remission rates rising to over 80% at follow‐up, the two studies that addressed the cognitive processes outlined in Dugas et al.’s (1998) model achieved impressive outcomes (Holmes et al., 2014; Perrin et al., 2019). Their results are considerably better than those reported across different anxiety disorders in previous meta‐analyses (e.g., James et al., 2020). Instead, they align more with the superior outcomes of targeted therapies for other youth anxiety disorders (e.g., 91% remission for adolescent social anxiety disorder at follow‐up; Leigh & Clark, 2023), and the meta‐analytic finding that targeted interventions are more effective than generic psychotherapies (Reynolds et al., 2012). This is tentative evidence for the increased efficacy of disorder‐specific over transdiagnostic treatments, with further support provided by non‐controlled studies—several of which have reported promising outcomes for GAD‐specific treatments (Esbjørn et al., 2018; Wahlund, Andersson, et al., 2020).

We identified a wealth of RCTs that assessed transdiagnostic treatments, but just three had data available for the subset of participants with primary GAD and an additional five provided results for mixed anxiety samples that did not differ by primary diagnosis. Whilst we can draw only provisional conclusions based on this limited amount of (variable quality) evidence, it appears that remission rates from primary GAD hover around 60%. This aligns with previous meta‐analyses (e.g., James et al., 2020), and may indicate that at long‐term assessment points, the targeted therapies assessed by Holmes et al. (2014) and Perrin et al. (2019) achieved higher remission rates compared to the disorder‐generic treatments. However, this is a preliminary inference; further research is needed to facilitate a robust comparison of disorder‐specific and transdiagnostic treatment approaches.

These findings support our call for greater disorder‐specific research practices, which would facilitate more detailed understanding of each anxiety diagnosis in its own right. Currently, it is a rarity within this field to analyse outcomes at the level of each disorder; therefore moving forwards, we urge researchers to take steps to make it possible to do so. Whilst it may not be informative for single studies to conduct diagnosis‐specific analyses due to low cell sizes, results can be made accessible on data archives and consequently combined across studies to increase statistical power (e.g., via initiatives such as the platform on anxiety disorders in youth (PADDY; Bertie et al., 2024)).

Firstly, researchers must maximise the quality and utility of the information that they record and share—in particular, they should capture putative mechanisms and markers of clinical change beyond simple remission or symptom change (Creswell et al., 2021). With respect to GAD, these may include measures of worry, depression, and cognitive processes (e.g., intolerance of uncertainty)—and importantly, results should be adequately powered and have a low risk of bias to reduce the risk of inflated effect sizes (Anderson et al., 2017). Secondly, studies included few adolescent and neurodiverse participants; this conflicts with the heightened levels of anxiety symptoms in these two groups (Accardo et al., 2024; NHS Digital, 2018; Waite & Creswell, 2014) and thus their representation must be considered in future. Of additional note, four of the six studies included in our synthesis made no mention of adverse events. This may mean that no such occurrences took place; however, in the absence of reporting, we are unable to rule out the possibility that adverse events occurred but were not recorded or reported. This oversight is common in clinical trials, particularly those using psychological interventions (Duggan et al., 2014), but must be addressed moving forwards to ensure the safety of treatments being delivered to vulnerable populations. Finally, whilst no trials conducted formal cost‐effectiveness analyses, all interventions required upwards of ten in‐person sessions. Thus, we may safely assume that their resource‐heavy nature could hamper implementation in over‐stretched services (Neufeld et al., 2017). Therefore, further work is needed to evaluate more efficient ways of disseminating equally effective therapies (see Wahlund, Jolstedt, et al. (2020) for a promising evaluation of an online intervention for adolescents with excessive worry).

### Strengths and limitations

This review is the first of its kind, with strengths including a reproducible search, independent double‐screening, and adherence to validated guidelines. Yet despite considerable effort, we were only able to obtain data for a small number of studies, many of which were deemed to be methodologically weak. It is also possible that our search was influenced by selection and language bias, and our inclusion criteria may have resulted in relevant evidence from non‐peer‐reviewed or non‐randomised studies being discarded. However, given the weaknesses of grey literature and non‐RCT designs, inclusion of such data may have added only confusion rather than clarity.

### Conclusion

Commonly misconceived as a less serious diagnosis (Newman & Przeworski, 2018), or the disorder of the ‘worried well’ (Ballenger et al., 2001), GAD is in fact a chronic and severe condition. However, few child and adolescent anxiety treatment studies have assessed this diagnosis in its own right. This review was the first to assess the efficacy of psychological therapies for youth with primary GAD—yet despite our best efforts to source data, available evidence is limited. Whilst preliminary results suggest that current psychological therapies may increase remission rates relative to no treatment, findings are far from conclusive and there is a clear need for improved research and reporting practices.

## AUTHOR CONTRIBUTIONS

**Lottie Shipp**: Conceptualisation; methodology; validation; formal analysis; investigation; resources; data curation; writing—original draft; writing—review and editing; visualisation; project administration. **Eleanor Leigh**: Conceptualisation; methodology; writing—review and editing; supervision. **Sakshi Rajesh**: Investigation. **Polly Waite**: Conceptualisation; methodology; writing—review and editing; supervision.

## CONFLICT OF INTEREST STATEMENT

The authors declare no conflicts of interest.

## ETHICS STATEMENT

This study is a systematic review, and no new data was collected, therefore no ethical approvals were required.

## Supporting information

Supporting Information S1

## Data Availability

Data sharing not applicable—No new data were created or analysed in this study.
