# Supplementary material for: Evaluating the efficacy of psychological therapies for generalised anxiety disorder in children and adolescents: A systematic review and narrative synthesis
Source: JCPP Adv. 2025 Oct 16;6(1):e70056. doi: 10.1002/jcv2.70056 (PMC12973129; doi:10.1002/jcv2.70056)
Supplement: Supplementary file 1 — Supporting Information S1 [file JCV2-6-e70056-s001.docx]

**Evaluating the efficacy of psychological therapies for Generalised Anxiety Disorder in children and adolescents: A systematic review and narrative synthesis**

Lottie Shipp, Eleanor Leigh*, Sakshi Rajesh, Polly Waite

Supporting Information

#### **Appendix S1.** Search strategy

|  | ***PsycINFO (OVID) (1806 to present)*** |
| --- | --- |
| 1 | anxiety disorders/ or exp generalized anxiety disorder/ |
| 2 | (“anxiety” or “anxious” or “GAD” or “worry”).ab. |
| 3 | 1 or 2 |
| 4 | (“adolescen*” or “youth*” or “teenage*” or “child*” or “p#edia*”).ab. |
| 5 | (“therap*” or “treatment*” or “sychotherapy*” or “intervention*” or “mindfulness” or “counselling” or “CBT” or “ACT” or “IPT” or “EFT”).ab. |
| 6 | randomized clinical trials/ or randomized controlled trials/ |
| 7 | “randomi?ed”.ab. |
| 8 | 6 or 7 |
| 9 | 3 and 4 and 5 and 8 |

|  | ***Embase (OVID) (1974 to present)*** |
| --- | --- |
| 1 | anxiety disorder/ or exp generalized anxiety disorder/ |
| 2 | ("anxiety" or "anxious" or "GAD" or "worry").ab. |
| 3 | 1 or 2 |
| 4 | ("adolescen*" or "youth*" or "teenage*" or "child*" or "pedia*" or "paedia*").ab. |
| 5 | randomized controlled trial/ or controlled clinical trial/ |
| 6 | "randomi?ed".ab. |
| 7 | 5 or 6 |
| 8 | ("therap*" or "treatment*" or "psychotherap*" or "intervention*" or "mindfulness" or "counselling" or "CBT" or "ACT" or "IPT" or "EFT").ab. |
| 9 | 3 and 4 and 7 and 8 |

|  | ***MedLine (OVID) (1946 to present)*** |
| --- | --- |
| 1 | Anxiety Disorders/ |
| 2 | ("anxiety" or "anxious" or "GAD" or "worry").ab. |
| 3 | 1 or 2 |
| 4 | ("adolescen*" or "youth*" or "teenage*" or "child*" or "p?edia*").ab. |
| 5 | ("therap*" or "treatment*" or "psychotherap*" or "intervention*" or "mindfulness" or "counselling" or "CBT" or "ACT" or "IPT" or "EFT").ab. |
| 6 | Randomized Controlled Trials as Topic/ |
| 7 | "randomi#ed".ab. |
| 8 | 6 or 7 |
| 9 | 3 and 4 and 5 and 8 |

|  | ***Cochrane Central Register of Controlled Trials*** |
| --- | --- |
| 1 | ("anxiety disorder"):ab (Word variations have been searched) with Publication Year from 1994 to 2024, with Cochrane Library publication date Between Jan 1994 and May 2024, in Trials |
| 2 | ("adolescent" or "youth" or "teenage" or "child" or "paediatric"):ab with Publication Year from 1994 to 2024, with Cochrane Library publication date Between Jan 1994 and May 2024, in Trials (Word variations have been searched) |
| 3 | ("therapy" or "psychotherapy" or "treatment" or "intervention" or "mindfulness" or "counselling" or "CBT" or "ACT" or "IPT" or "EFT"):ab with Publication Year from 1994 to 2024, with Cochrane Library publication date Between Jan 1994 and May 2024, in Trials (Word variations have been searched) |
| 4 | MeSH descriptor: [Randomized Controlled Trial] explode all trees |
| 5 | ("randomised" or "randomized"):ab |
| 6 | #4 OR #5 |
| 7 | #1 AND #2 AND #3 AND #6 |

| ***ClinicalTrials.gov*** | |
| --- | --- |
| ***Search 1*** | |
| Condition/disease | Generalised Anxiety Disorder |
| Other terms | Children |
| Intervention/treatment | - |
| Location | - |
| ***Search 1*** | |
| Condition/disease | Generalised Anxiety Disorder |
| Other terms | Adolescents |
| Intervention/treatment | - |
| Location | - |

***Appendix S2.*** *Hierarchy of measures*

**Worry**

|  | **Measure** | **Notes** |
| --- | --- | --- |
| 1. | Penn State Worry Questionnaire for Children (**PSWQ-C**; Chorpita et al., 1997) | Chosen as the preferred outcome measure as it is a) widely used, b) designed for children aged 6-18 years, and c) has been validated in community and clinical samples (e.g., Chorpita et al., 1997; Pestle et al., 2008) . |

***Child-reported outcomes will be prioritised over parent-reported outcomes. Where only parent data are provided, the above hierarchy of measures will be followed.***

**Anxiety**

|  | **Measure** | **Broad anxiety or GAD-specific** | **Rater** | **Notes** |
| --- | --- | --- | --- | --- |
| 1. | Clinician Severity Rating (**CSR**) for Generalised Anxiety Disorder | GAD-specific | Clinician | Clinician rated severity of GAD symptoms (part of ADIS interview). Chosen as the preferred outcome measure, given that structured diagnostic interviews are considered the gold-standard assessment approach. |
| 2. | Paediatric Anxiety Rating Scale (**PARS**; (The Research Units On Pediatric Psychopharmacology Anxiety Study Group, 2002) | GAD-specific | Clinician | Rated highly as this is a clinician-report measure. It is used to assess the severity of DSM-IV anxiety disorders. It has good inter-rater reliability, adequate test-retest reliability, and fair internal consistency (The Research Units On Pediatric Psychopharmacology Anxiety Study Group, 2002) . It has shown convergent validity with SCARED, and good sensitivity and specificity (Ginsburg et al., 2011) . |
| 3. | Clinical Global Impressions Scales – Severity (**CGI-Severity**) | Broad anxiety/GAD specific | Clinician | Clinician-rated severity of overall psychopathology (although some studies use these ratings for target disorders). CGI was developed as a measure of pre- to post-treatment change in severity/functioning in NIMH clinical trials. It is rated lower than CSR and PARS given that it is only one question (“Considering your total clinical experience with this particular population, how mentally ill is the patient at this time?”), with ratings given on a scale from 1-7 (Dunlop et al., 2017) . |
| 2. | Spence Children’s Anxiety Scale – GAD subscale (**SCAS-GAD**; Spence, 1997) | GAD-specific | Child/adolescent | Rated favourably as it a) was developed specifically for children, b) has been used widely in research and clinical settings, and c) has been shown to be sensitive to treatment (Myers & Winters, 2002) . It has a subscale that maps onto the DSM-IV criteria for GAD, in addition to panic disorder, separation anxiety disorder, social phobia, and school refusal subscales. A meta-analysis revealed that the GAD scale has high reliability (alpha value; Hale et al. (2011) ). |
| 3. | Screen for Child Anxiety Related Disorders – GAD subscale (**SCARED-GAD**; (Birmaher et al., 1997) | GAD-specific | Child/adolescent | SCARED has five subscales: generalised anxiety, separation anxiety, social anxiety, panic, and school avoidance. It is rated below SCAS as it seems to be used less frequently in RCTs. There is evidence for good psychometric properties and sensitivity to treatment (Spence, 2018) . |
| 4. | Revised Child Anxiety and Depression Scale – GAD subscale (**RCADS-GAD**; Chorpita et al., 2000) | GAD-specific | Child/adolescent | This is rated below the SCAS-GAD and SCARED-GAD in the hierarchy, as items do not cover the entire breadth of the DSM-V diagnostic criteria for GAD (“I worry about things”; “I worry that something awful will happen to someone in my family”; “I worry that bad things will happen to me”; “I worry that something bad will happen to me”; “I worry about what is going to happen”; and “I think about death”). |
| 5. | **GAD-7** (Spitzer et al., 2006) | GAD-specific | Child/adolescent | This measure is further down the hierarchy, as it was not developed/adapted for children and adolescents. It has been used with adolescent samples (Beard & Björgvinsson, 2014; Guzick et al., 2024), but not widely with younger children. |
| 6. | Spence Children’s Anxiety Scale – total anxiety score (**SCAS-total**; Spence, 1998) | Broad anxiety | Child/adolescent | This broad measure of overall anxiety shows good internal consistency, which significantly correlates with SCARED to show convergent validity (Muris et al., 2000). |
| 7. | Screen for Child Anxiety Related Disorders – total score (**SCARED-total**; (Birmaher et al., 1997) | Broad anxiety | Child/adolescent | SCARED total score has good psychometric properties (Birmaher et al., 1997). It is rated below the SCAS only as seems to be used less frequently. |
| 8. | Multidimensional Anxiety Scale for Children – total score (**MASC-total**; March et al., 1997) | Broad anxiety | Child/adolescent | The MASC is positioned lower down the hierarchy as it is not GAD-specific (given that it consists of four subscales: physical symptoms, harm avoidance, social anxiety and separation anxiety). Scales can be indirectly related to diagnoses, but do not directly map onto the DSM-V criteria. Therefore, the MASC is more appropriate for the measurement of broad anxiety. It has been shown to be superior to the RCMAS (Myers & Winters, 2002). |
| 9. | Revised Children’s Manifest Anxiety Scale – total score (**RCMAS**; Reynolds & Richmond, 1978) | Broad anxiety | Child/adolescent | The RCMAS measures chronic manifestations of trait anxiety (physiological anxiety, worry/oversensitivity, fear/concentration, lie (social desirability)). It correlates with the STAI-C but appears to have poor discriminant validity and has a dichotomous yes/no response format which may reduce its sensitivity and power (Myers & Winters, 2002). This measure is less accurate than multidimensional measures such as SCARED (Spence, 2018). It can discriminate between youth with and without anxiety disorders, although it is less useful for distinguishing between anxiety disorders and affective disorders (Seligman et al., 2004). 21% of the RCMAS items overlap with Children’s Depression Inventory Items (Seligman & Ollendick, 1998); therefore, it may be measuring depression in addition to anxiety. |
| 10. | State-Trait Anxiety Inventory for Children – trait subscale (**STAIC-Trait;**  Spielberger et al. (1973) ) | Broad anxiety | Child/adolescent | This is a general anxiety measure that can discriminate between youth with and without anxiety disorders (Seligman et al., 2004). However, discriminant validity is limited (25% of items on the STAIC-Trait subscale overlap with items of the Children’s Depression Inventory (Seligman & Ollendick, 1998)). |

***Clinician and child-reported outcomes will be prioritised over parent-reported outcomes. Where only parent data are provided, the above hierarchy of measures will be followed.***

**Depression**

|  | **Measure** | **Rater** | **Notes** |
| --- | --- | --- | --- |
| 1. | Clinician Severity Rating (**CSR**) for MDD | Clinician | This is the preferred outcome measure as it is the gold-standard clinical assessment used widely in research. |
| 2. | Hamilton Rating Scale for Depression (**HRSD**; Hamilton, 1960) | Clinician | HRSD is used less frequently than CSR, and therefore ranks second in the hierarchy. However, it does have excellent psychometric properties - although stability has not been widely evaluated (Myers & Winters, 2002) and it is not youth-specific (Simmons et al., 2015). |
| 3. | Mood and Feelings Questionnaire (**MFQ**; Costello & Angold, 1988) | Child/adolescent | The MFQ contains 33 items and is based on DSM-IV criteria for MDD (i.e., it assesses depressive symptoms over the past two weeks on a 3-point scale). It has been widely used in research settings and has excellent internal consistency (Eg et al., 2018). It is rated relatively high up in the hierarchy as it has good diagnostic reliability and is recommended by NICE (Simmons et al., 2015). |
| 4. | Short Mood and Feelings Questionnaire (**SMFQ;** Angold et al., 1995) | Child/adolescent | A brief version of the MFQ. The SMFQ has been used for children and adolescents, and has been shown to have good discriminatory ability relevant to ICD-10 criteria for MDD (Turner et al., 2014). It is rated below the MFQ as it does not assess suicidal ideation (Simmons et al., 2015). |
| 5. | Children’s Depression Inventory (**CDI;**  Kovacs, 1978) | Child/adolescent | A modification of the adult BDI. It includes 27 items assessing the cognitive, affective, and behavioural symptoms of depression over the past 2 weeks. It has good discriminant validity between anxiety and depressive disorders relative to KID-SCID outcomes (Timbremont et al., 2004). However, it is rated lower than the MFQ and RCADS as it has a 3-point response format that may not be highly sensitive to treatment effects (Myers & Winters, 2002), and Simmons et al. (2015) suggest that it does not adequately distinguish between depressed and non-depressed youths. |
| 6. | Revised Child Anxiety and Depression Scale – Depression subscale (**RCADS-Depression;**  Chorpita et al., 2000) | Child/adolescent | RCADS is widely used in clinical services in the UK (Law & Wolpert, 2014) and is also used in research settings. It maps onto the DSM-IV criteria for GAD, SP, SAD, OCD, PD, and MDD. It has good internal consistency and short-term stability (but low agreement between the RCADS depression subscale and the Problem Behavior at School Inventory depression subscale). Additionally, high correlations between the anxiety and depression subscales indicates that the MDD scale may be tapping into anxiety (Kösters et al., 2015), and it may not be able to distinguish between mood disorders (Simmons et al., 2015). Therefore, it is ranked below the MFQ and SMFQ. |
| 7. | Center for Epidemiologic Studies – Depression Scale for Children and adolescents (**CES-DC;**  Weissman et al., 1980) | Child/adolescent | The CES-DC assesses symptoms of depression over the past week, and is widely used. It has good internal reliability and moderate stability in adolescent populations, but poorer psychometric properties for children and boys (Myers & Winters, 2002). It is rated lower down the hierarchy as it is suggested to capture general psychopathology as opposed to depression more specifically (Simmons et al., 2015). |
| 8. | Reynolds Adolescent Depression Scale / Reynolds Child Depression Scale (Reynolds, 1989, 2002) | Child/adolescent | Based on DSM-III-R criteria for MDD. Despite good internal consistency, it is rated lower in the hierarchy as it was developed for school samples, has limited ability to detect change in symptoms, and appears to have a high false positive rate (Simmons et al., 2015). |

***Clinician and child-reported outcomes will be prioritised over parent-reported outcomes. Where only parent data are provided, the above hierarchy of measures will be followed.***

***Appendix S3****. ORBIT Matrix*

| *Study* | *Primary outcomes* | *Secondary outcomes* | | | | | | | | | | | | | | | | | | | | | | |
| --- | --- | --- | --- | --- | --- | --- | --- | --- | --- | --- | --- | --- | --- | --- | --- | --- | --- | --- | --- | --- | --- | --- | --- | --- |
|  | *GAD diagnosis* | *Anxiety* | *Worry* | *Depression* | *Conduct problems* | *Peer difficulties* | *Global functioning* | *Quality of life* | *IU* | *CA* | *NPO* | *PBW* | *NBW* | *Treatment credibility* | *Therapeutic engagement* | *Internalising* | *Perfectionism* | *Treatment satisfaction* | *Sleep* | *Social anxiety* | *Somatic complaints* | *Externalising* | *Social withdrawal* |  |
| *Perrin (2019)* | *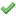* | *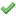* | *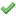* | *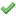* | *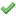* | *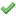* | *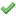* | *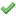* | *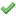* | *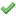* | *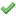* | *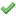* | *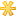* | *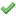* | *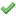* | *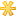* | *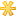* | *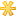* | *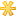* | *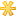* | *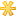* | *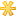* | *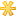* |  |
| *Holmes (2014)* | *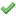* | *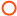* | *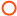* | *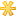* | *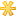* | *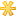* | *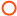* | *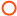* | *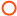* | *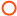* | *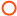* | *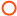* | *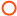* | *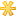* | *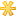* | *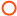* | *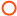* | *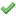* | *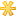* | *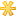* | *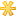* | *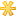* | *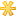* |  |
| *Clementi (2020)* | *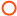* | *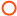* | *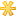* | *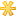* | *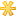* | *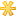* | *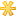* | *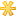* | *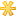* | *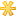* | *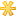* | *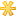* | *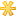* | *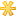* | *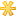* | *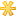* | *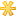* | *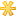* | *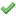* | *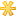* | *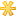* | *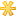* | *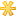* |  |
| *Goldbeck (2012)* | *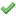* | *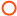* | *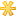* | *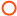* | *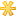* | *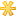* | *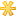* | *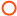* | *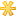* | *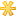* | *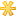* | *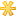* | *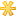* | *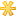* | *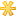* | *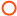* | *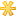* | *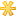* | *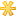* | *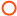* | *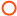* | *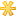* | *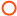* |  |
| *Wergeland (2014)* | *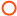* | *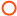* | *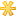* | *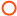* | *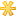* | *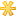* | *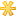* | *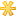* |  |  |  |  |  |  |  |  |  |  |  |  |  |  |  |  |
| *Villabø (2018)* |  |  |  |  |  |  |  |  |  |  |  |  |  |  |  |  |  |  |  |  |  |  |  |  |

*NOTE: Full reporting was defined as sufficient data provided for inclusion in a statistical synthesis without the need for additional contact with study authors. Partial reporting was defined as reporting either a) completer data (raw scores) only for participants with primary GAD with no ITT data, or b) reporting ITT or completer data for a mixed anxiety sample. This table includes only child symptoms (parent anxiety was measured in several studies but not included here). Green tick = full reporting; yellow star = not measured; red circle = partial reporting. IU = intolerance of uncertainty; CA = cognitive avoidance; NPO = negative problem orientation; PBW = positive beliefs about worry; NBW = negative beliefs about worry.*

***Table S1.*** *Studies reporting no moderation of treatment outcomes by primary diagnosis*

| **Study** | **Psychological therapy** | **Control condition** | **Risk of bias** | **Age (mean, range)** | **Total n** | **n with primary GAD (%)** | **Treatment components (child sessions)** | **Therapy dose and duration** | **Clinician background** | **Parental involvement** | **Treatment outcome** | **Log odds ratio of remission from all anxiety disorders [SE]** |
| --- | --- | --- | --- | --- | --- | --- | --- | --- | --- | --- | --- | --- |
| Shortt (2001) | Group CBT | WLC | High | 7.9 years (6-10 years) | 71 | 42 (59%) | Psychoeducation, cognitive restructuring, self-monitoring, relaxation training, developing a social network, using coping self-talk, rewarding oneself, and attentional training. | 10 weekly sessions for children and 2 booster sessions (50-60 mins each). 10 separate parent sessions (40 mins each). Up to 18 hours of therapist time per family. | Clinical masters-trained doctoral psychologists. | Ten separate parent sessions. | Authors report that “no significant difference was found between the diagnostic groups at post-treatment”. In the treatment group, n = 33 (61%) were in remission at post-treatment relative to n = 2 (12%) of those in the WLC group. | 2.47 [0.64] |
| Barrington (2005) | Individual CBT | TAU | Some concerns | 10.0 years (7-14 years) | 54 | 12 (22%) | Psychoeducation regarding anxiety-related thoughts and feelings, cognitive restructuring and graded exposure to anxiety-provoking situations. | 12 weekly sessions (no duration given). | Master’s or doctoral level psychologists with at least 2 years’ postgraduate training in CBT. | Sessions involved parents at the discretion of the therapist. | Participants with primary social phobia at baseline had the poorest treatment outcomes. Authors report that “otherwise, treatment outcomes appeared unaffected by pre-treatment group differences in the frequency of primary diagnoses”. Removing participants with primary social phobia (*n* = 9, leaving *n* = 45 in the sample) results in a remission rate from all anxiety disorders of 84% at 12-month follow-up in the CBT group, and 75% remission in the TAU group. | 0.56 [0.56] |
| Cobham (1998) | Group child-focused CBT plus parental anxiety management | Group child-focused CBT | Some concerns | 9.6 years (7-14 years) | 67 | 40 (60%) | Cognitive restructuring, exposure, relaxation training, introducing coping self-talk, and use of rewards. | 10 weekly sessions for children with an additional 4 parent sessions (60 mins per session). Total of 14 hours of therapist time per family. | Doctoral-level psychologist (plus co-therapist in 40% of groups). | 4 separate parent sessions (involving psychoeducation about child anxiety and strategies for parents to manage their own anxiety). | 78% of participants in the treatment condition were in remission from all anxiety disorders at post-treatment compared to 60% remission in control group. Authors report that there was no effect of initial diagnosis on outcome. | 0.87 [0.30] |
| Silk (2018) | Individual CBT | Child-centred therapy | Low | 10.9 years (9-14 years) | 133 | 94 (71%) | Psychoeducation, cognitive restructuring, exposure, self-monitoring, relaxation training, coping self-talk and use of rewards. | 14 sessions with children and 2 parent sessions. | Master’s or doctoral level psychologists. | Two parent-only sessions. | 67% of the treatment group were in full recovery (i.e., no anxiety disorder diagnoses) at post-treatment compared to 47% of control group. Anxiety subtype did not predict or moderate treatment response. | 0.83 [0.14] |
| Suveg (2018) | Emotion-focused CBT | CBT (Coping Cat) | Low | 8.9 years (7-12 years) | 92 | 50 (54%) | Psychoeducation about anxiety and emotions (guilt, sadness, anger, etc), cognitive restructuring, exposure, self-monitoring, relaxation training, and challenging difficult emotions. | 10 weekly sessions (1 hour each). Total of 10 hours of therapist time per family. | Graduate clinical psychology doctoral students. | Parents included in two of the child’s sessions. | 35% of children in the intervention group were in remission from all anxiety disorders at post-treatment, compared to 47% in the control group. Authors report that “there were no differences in treatment outcomes by primary diagnosis”. Remission from the primary anxiety disorder was reported for 55% and 60% of participants in the treatment and control conditions, respectively. | -0.49 [0.18] |

***Appendix S4.*** *PRISMA checklist*

| **Section and Topic** | **Item #** | **Checklist item** | **Location where item is reported** |
| --- | --- | --- | --- |
| **TITLE** | | |  |
| Title | 1 | Identify the report as a systematic review. | Title |
| **ABSTRACT** | | |  |
| Abstract | 2 | See the PRISMA 2020 for Abstracts checklist. | See below |
| **INTRODUCTION** | | |  |
| Rationale | 3 | Describe the rationale for the review in the context of existing knowledge. | ‘Current gaps in understanding’ |
| Objectives | 4 | Provide an explicit statement of the objective(s) or question(s) the review addresses. | ‘Research questions’ |
| **METHODS** | | |  |
| Eligibility criteria | 5 | Specify the inclusion and exclusion criteria for the review and how studies were grouped for the syntheses. | Table 1 and ‘Study characteristics’ |
| Information sources | 6 | Specify all databases, registers, websites, organisations, reference lists and other sources searched or consulted to identify studies. Specify the date when each source was last searched or consulted. | ‘Methods’ |
| Search strategy | 7 | Present the full search strategies for all databases, registers and websites, including any filters and limits used. | Supporting Information (S1) |
| Selection process | 8 | Specify the methods used to decide whether a study met the inclusion criteria of the review, including how many reviewers screened each record and each report retrieved, whether they worked independently, and if applicable, details of automation tools used in the process. | Fig 1 and ‘Results’ |
| Data collection process | 9 | Specify the methods used to collect data from reports, including how many reviewers collected data from each report, whether they worked independently, any processes for obtaining or confirming data from study investigators, and if applicable, details of automation tools used in the process. | ‘Data extraction’ |
| Data items | 10a | List and define all outcomes for which data were sought. Specify whether all results that were compatible with each outcome domain in each study were sought (e.g. for all measures, time points, analyses), and if not, the methods used to decide which results to collect. | ‘Data extraction’ and Supplementary Information (S4) |
|  | 10b | List and define all other variables for which data were sought (e.g. participant and intervention characteristics, funding sources). Describe any assumptions made about any missing or unclear information. | ‘Data extraction’ |
| Study risk of bias assessment | 11 | Specify the methods used to assess risk of bias in the included studies, including details of the tool(s) used, how many reviewers assessed each study and whether they worked independently, and if applicable, details of automation tools used in the process. | ‘Risk of bias’ |
| Effect measures | 12 | Specify for each outcome the effect measure(s) (e.g. risk ratio, mean difference) used in the synthesis or presentation of results. | ‘Data synthesis and analysis’ |
| Synthesis methods | 13a | Describe the processes used to decide which studies were eligible for each synthesis (e.g. tabulating the study intervention characteristics and comparing against the planned groups for each synthesis (item #5)). | ‘Data synthesis and analysis’ |
|  | 13b | Describe any methods required to prepare the data for presentation or synthesis, such as handling of missing summary statistics, or data conversions. | ‘Data extraction’ and ‘Results’ |
|  | 13c | Describe any methods used to tabulate or visually display results of individual studies and syntheses. | ‘Study characteristics’ |
|  | 13d | Describe any methods used to synthesize results and provide a rationale for the choice(s). If meta-analysis was performed, describe the model(s), method(s) to identify the presence and extent of statistical heterogeneity, and software package(s) used. | ‘Data synthesis and analysis’ |
|  | 13e | Describe any methods used to explore possible causes of heterogeneity among study results (e.g. subgroup analysis, meta-regression). | NA |
|  | 13f | Describe any sensitivity analyses conducted to assess robustness of the synthesized results. | NA |
| Reporting bias assessment | 14 | Describe any methods used to assess risk of bias due to missing results in a synthesis (arising from reporting biases). | ‘Risk of bias’ |
| Certainty assessment | 15 | Describe any methods used to assess certainty (or confidence) in the body of evidence for an outcome. | ‘Risk of bias’ |
| **RESULTS** | | |  |
| Study selection | 16a | Describe the results of the search and selection process, from the number of records identified in the search to the number of studies included in the review, ideally using a flow diagram. | Fig 1 |
|  | 16b | Cite studies that might appear to meet the inclusion criteria, but which were excluded, and explain why they were excluded. | ‘Results’ |
| Study characteristics | 17 | Cite each included study and present its characteristics. | ‘Study characteristics’ and Table 2 |
| Risk of bias in studies | 18 | Present assessments of risk of bias for each included study. | Fig 3 |
| Results of individual studies | 19 | For all outcomes, present, for each study: (a) summary statistics for each group (where appropriate) and (b) an effect estimate and its precision (e.g. confidence/credible interval), ideally using structured tables or plots. | Table 2 and Fig 2 |
| Results of syntheses | 20a | For each synthesis, briefly summarise the characteristics and risk of bias among contributing studies. | ‘Results’, ‘Risk of bias’, and Fig 3 |
|  | 20b | Present results of all statistical syntheses conducted. If meta-analysis was done, present for each the summary estimate and its precision (e.g. confidence/credible interval) and measures of statistical heterogeneity. If comparing groups, describe the direction of the effect. | Table 2 |
|  | 20c | Present results of all investigations of possible causes of heterogeneity among study results. | NA |
|  | 20d | Present results of all sensitivity analyses conducted to assess the robustness of the synthesized results. | NA |
| Reporting biases | 21 | Present assessments of risk of bias due to missing results (arising from reporting biases) for each synthesis assessed. | ‘Risk of bias’ and Fig 3 |
| Certainty of evidence | 22 | Present assessments of certainty (or confidence) in the body of evidence for each outcome assessed. | ‘Certainty assessment’ |
| **DISCUSSION** | | |  |
| Discussion | 23a | Provide a general interpretation of the results in the context of other evidence. | ‘Discussion’ |
|  | 23b | Discuss any limitations of the evidence included in the review. | ‘Strengths and limitations’ |
|  | 23c | Discuss any limitations of the review processes used. | ‘Strengths and limitations’ |
|  | 23d | Discuss implications of the results for practice, policy, and future research. | ‘A call for improvements to research and reporting practices’ |
| **OTHER INFORMATION** | | |  |
| Registration and protocol | 24a | Provide registration information for the review, including register name and registration number, or state that the review was not registered. | ‘Methods’ |
|  | 24b | Indicate where the review protocol can be accessed, or state that a protocol was not prepared. | ‘Methods’ |
|  | 24c | Describe and explain any amendments to information provided at registration or in the protocol. | NA |
| Support | 25 | Describe sources of financial or non-financial support for the review, and the role of the funders or sponsors in the review. | ‘Acknowledgements’ |
| Competing interests | 26 | Declare any competing interests of review authors. | ‘Acknowledgements’ |
| Availability of data, code and other materials | 27 | Report which of the following are publicly available and where they can be found: template data collection forms; data extracted from included studies; data used for all analyses; analytic code; any other materials used in the review. | ‘Acknowledgements’ |

*From:*  Page MJ, McKenzie JE, Bossuyt PM, Boutron I, Hoffmann TC, Mulrow CD, et al. The PRISMA 2020 statement: an updated guideline for reporting systematic reviews. BMJ 2021;372:n71. doi: 10.1136/bmj.n71

***PRISMA - extension for abstracts***

| **Section and Topic** | **Item #** | **Checklist item** | **Reported (Yes/No)** |
| --- | --- | --- | --- |
| **TITLE** | | |  |
| Title | 1 | Identify the report as a systematic review. | Y |
| **BACKGROUND** | | |  |
| Objectives | 2 | Provide an explicit statement of the main objective(s) or question(s) the review addresses. | Y |
| **METHODS** | | |  |
| Eligibility criteria | 3 | Specify the inclusion and exclusion criteria for the review. | Y |
| Information sources | 4 | Specify the information sources (e.g. databases, registers) used to identify studies and the date when each was last searched. | Y |
| Risk of bias | 5 | Specify the methods used to assess risk of bias in the included studies. | Y |
| Synthesis of results | 6 | Specify the methods used to present and synthesise results. | Y |
| **RESULTS** | | |  |
| Included studies | 7 | Give the total number of included studies and participants and summarise relevant characteristics of studies. | Y |
| Synthesis of results | 8 | Present results for main outcomes, preferably indicating the number of included studies and participants for each. If meta-analysis was done, report the summary estimate and confidence/credible interval. If comparing groups, indicate the direction of the effect (i.e. which group is favoured). | Y |
| **DISCUSSION** | | |  |
| Limitations of evidence | 9 | Provide a brief summary of the limitations of the evidence included in the review (e.g. study risk of bias, inconsistency and imprecision). | Y |
| Interpretation | 10 | Provide a general interpretation of the results and important implications. | Y |
| **OTHER** | | |  |
| Funding | 11 | Specify the primary source of funding for the review. | Y (Acknowledgements) |
| Registration | 12 | Provide the register name and registration number. | Y |

*From:* Page MJ, McKenzie JE, Bossuyt PM, Boutron I, Hoffmann TC, Mulrow CD, et al. The PRISMA 2020 statement: an updated guideline for reporting systematic reviews. BMJ 2021;372:n71. doi: 10.1136/bmj.n71.

**S*6:*** *SWiM checklist*

| **SWiM is intended to complement and be used as an extension to PRISMA** | | | |
| --- | --- | --- | --- |
| **Reporting item** | **Item description** | **Page in manuscript where item is reported** | **Other*** |
| ***Methods*** | | | |
| **1** Grouping studies for synthesis | 1a) Provide a description of, and rationale for, the groups used in the synthesis (e.g., groupings of populations, interventions, outcomes, study design) | p.9 |  |
|  | 1b) Detail and provide rationale for any changes made subsequent to the protocol in the groups used in the synthesis | NA |  |
| **2** Describe the standardised metric and transformation methods used | Describe the standardised metric for each outcome. Explain why the metric(s) was chosen, and describe any methods used to transform the intervention effects, as reported in the study, to the standardised metric, citing any methodological guidance consulted | p.8 |  |
| **3** Describe the synthesis methods | Describe and justify the methods used to synthesise the effects for each outcome when it was not possible to undertake a meta-analysis of effect estimates | p.8 |  |
| **4** Criteria used to prioritise results for summary and synthesis | Where applicable, provide the criteria used, with supporting justification, to select the particular studies, or a particular study, for the main synthesis or to draw conclusions from the synthesis (e.g., based on study design, risk of bias assessments, directness in relation to the review question) | p.6 |  |
| **5** Investigation of heterogeneity in reported effects | State the method(s) used to examine heterogeneity in reported effects when it was not possible to undertake a meta-analysis of effect estimates and its extensions to investigate heterogeneity | NA |  |
| **6** Certainty of evidence | Describe the methods used to assess certainty of the synthesis findings | p.8-9 |  |
| **7** Data presentation methods | Describe the graphical and tabular methods used to present the effects (e.g., tables, forest plots, harvest plots).  Specify key study characteristics (e.g., study design, risk of bias) used to order the studies, in the text and any tables or graphs, clearly referencing the studies included | p.9 |  |
| ***Results*** | | | |
| **8** Reporting results | For each comparison and outcome, provide a description of the synthesised findings, and the certainty of the findings. Describe the result in language that is consistent with the question the synthesis addresses, and indicate which studies contribute to the synthesis | p.8-14 |  |
| ***Discussion*** |  |  |  |
| **9** Limitations of the synthesis | Report the limitations of the synthesis methods used and/or the groupings used in the synthesis, and how these affect the conclusions that can be drawn in relation to the original review question | p.16 |  |

PRISMA=Preferred Reporting Items for Systematic Reviews and Meta-Analyses.

*If the information is not provided in the systematic review, give details of where this information is available (e.g., protocol, other published papers (provide citation details), or website (provide the URL)).

*From:* Campbell M, McKenzie JE, Sowden A, Katikireddi SV, Brennan SE, Ellis S, Hartmann-Boyce J, Ryan R, Shepperd S, Thomas J, Welch V, Thomson H. Synthesis without meta-analysis (SWiM) in systematic reviews: reporting guideline BMJ 2020;368:l6890 <http://dx.doi.org/10.1136/bmj.l6890>
